# The Arbovirus Mapping and Prediction (ArboMAP) system for West Nile virus forecasting

**DOI:** 10.1093/jamiaopen/ooad110

**Published:** 2023-12-21

**Authors:** Dawn M Nekorchuk, Anita Bharadwaja, Sean Simonson, Emma Ortega, Caio M B França, Emily Dinh, Rebecca Reik, Rachel Burkholder, Michael C Wimberly

**Affiliations:** Department of Geography and Environmental Sustainability, University of Oklahoma, Norman, OK 73019, United States; South Dakota Department of Health, Pierre, SD 57501, United States; Louisiana Department of Health, New Orleans, LA 70112, United States; Louisiana Department of Health, New Orleans, LA 70112, United States; Department of Biology, Southern Nazarene University, Bethany, OK 73008, United States; Quetzal Education and Research Center, Southern Nazarene University, San Gerardo de Dota, 11911, Costa Rica; Michigan Department of Health and Human Services, Lansing, MI 48909, United States; Michigan Department of Health and Human Services, Lansing, MI 48909, United States; Michigan Department of Health and Human Services, Lansing, MI 48909, United States; Department of Geography and Environmental Sustainability, University of Oklahoma, Norman, OK 73019, United States

**Keywords:** mosquito, weather, surveillance, software, outbreak

## Abstract

**Objectives:**

West Nile virus (WNV) is the most common mosquito-borne disease in the United States. Predicting the location and timing of outbreaks would allow targeting of disease prevention and mosquito control activities. Our objective was to develop software (ArboMAP) for routine WNV forecasting using public health surveillance data and meteorological observations.

**Materials and Methods:**

ArboMAP was implemented using an R markdown script for data processing, modeling, and report generation. A Google Earth Engine application was developed to summarize and download weather data. Generalized additive models were used to make county-level predictions of WNV cases.

**Results:**

ArboMAP minimized the number of manual steps required to make weekly forecasts, generated information that was useful for decision-makers, and has been tested and implemented in multiple public health institutions.

**Discussion and Conclusion:**

Routine prediction of mosquito-borne disease risk is feasible and can be implemented by public health departments using ArboMAP.

## Background and significance

Diseases caused by mosquito-transmitted arboviruses are a global health threat. In the United States, West Nile virus (WNV) is the most common mosquito-borne disease. This virus is transmitted primarily by mosquitoes in the genus *Culex*, and wild birds are the zoonotic reservoir hosts.^1^ Most human infections are asymptomatic or cause only mild symptoms, but ∼25% cause West Nile fever and <1% result in severe neuroinvasive disease that can be fatal.^2^ The burden of human WNV disease is highly variable. In the conterminous United States between 2009 and 2018, total annual cases ranged from 712 to 5674 and average annual incidence of WNV neuroinvasive disease varied from 0.02 cases/100 000 in Maine to 3.16 cases/100 000 in North Dakota.^3^ Public health responses to WNV include prevention messaging to encourage behaviors that prevent mosquito bites and vector control activities to reduce vector abundance.^4^ Prediction of WNV outbreaks would allow proactive targeting of disease prevention and mosquito control activities to reduce transmission.

WNV surveillance commonly involves trapping and testing of vector mosquitoes, and the presence of WNV-infected mosquitoes is a strong indicator of the local risk of human disease.^5^ The vectors and hosts of WNV are also sensitive to habitat availability, and WNV cases exhibit lagged responses to meteorological factors such as temperature and humidity.^6–8^ Mosquito infection rates and environmental variables have been used to develop predictive models to forecast human cases throughout the transmission season. These models accurately predict seasonal outbreaks early enough in the year to allow public health responses prior to the annual peak in cases.^9–11^ However, many public health agencies lack the software and expertise that is needed to implement disease forecasting.

## Objectives

The objective of this project was to develop and implement the Arbovirus Mapping and Prediction (ArboMAP) software for WNV forecasting by epidemiologists working in state health departments in the United States.

## Methods

### System overview

**Figure 1.**
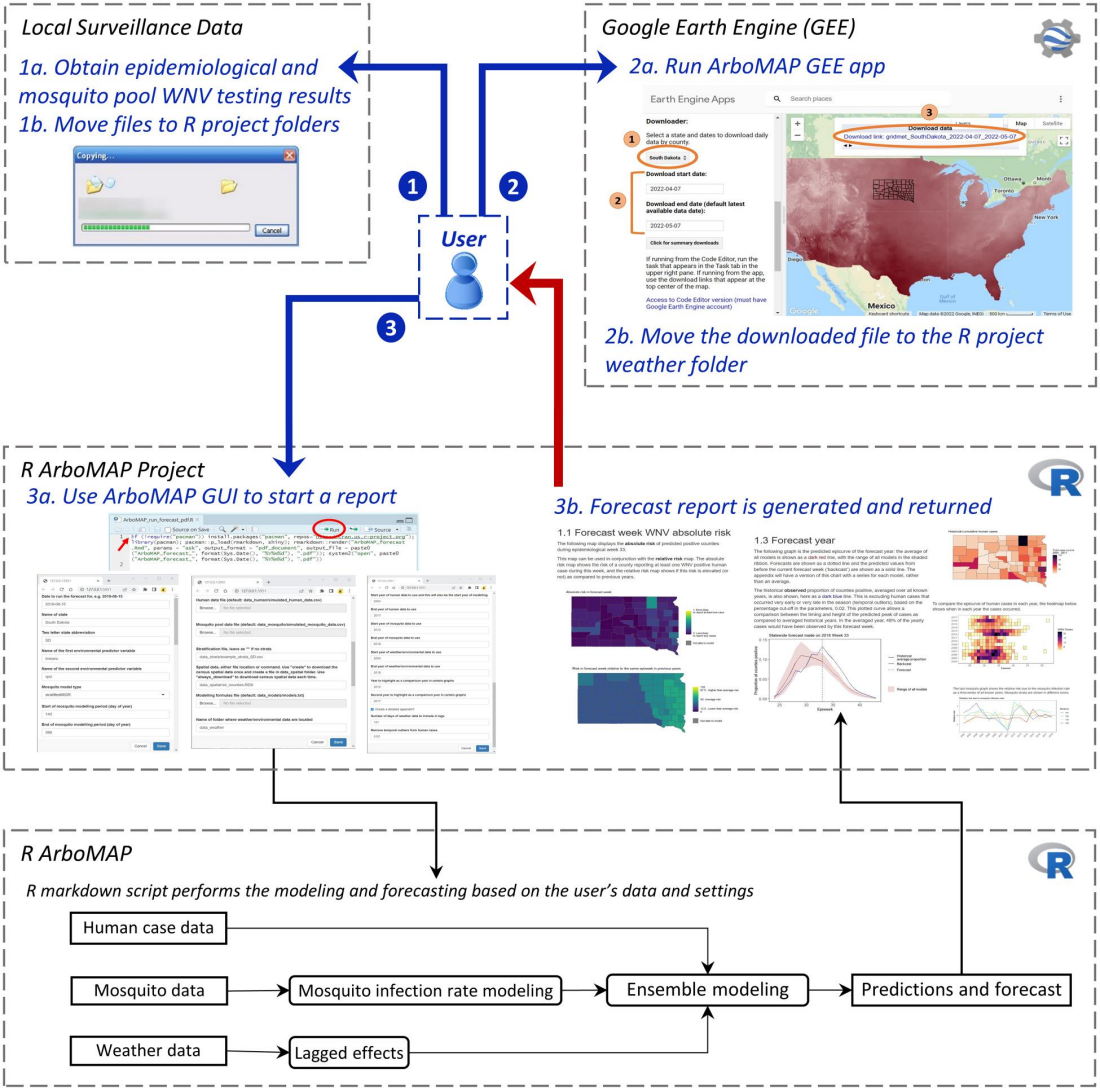
User-centered diagram showing the workflow for WNV forecasting. Step 1: Acquire updated entomological surveillance data, Step 2: Use the GEE app to update meteorological data, and Step 3: Use the RStudio GUI to generate a report. The system diagram on the bottom shows the high-level processes for modeling and forecasting.

ArboMAP is implemented in the R programming language using the RStudio interactive development environment with all code stored in an R Markdown script. The forecasting process begins with ingestion of new data and harmonization of multiple data sources into a unified format suitable for modeling (Figure 1). Models are calibrated using data from prior years, and recent observations are used to inform predictions of WNV cases during the current transmission season. A report containing summaries of the data and the forecasts is automatically generated. Forecasts are usually made for all counties within a US state and are produced by an epidemiologist or other public health professional working in a government agency that conducts vector-borne disease surveillance.

### Input data

Three sources of data are required. The first is de-identified human case data from surveillance databases, with each case referenced by the date of symptom onset and the county of residence. These data are converted to a weekly indicator variable for the occurrence of one or more human cases in each county. The second data source is mosquito testing results, also from surveillance databases. These data include one record for each pool of mosquitoes tested referenced by test result (positive or negative), the date of collection, and the county of collection. ArboMAP calculates indices of mosquito infection from these data, including the mosquito infection growth rate, which has been shown to be an effective predictor of human WNV cases in South Dakota.^10^ The third data source includes environmental variables that fluctuate throughout the transmission season. These data can be county-level summaries of daily meteorological variables such as temperature, humidity, precipitation, and windspeed or remotely sensed variables such as land surface temperature and spectral indices.^12^

To facilitate access to meteorological data, we developed a version of the Retrieving Environmental Analytics for Climate and Health (REACH) app^13^ to access meteorological data for WNV forecasting. We used the gridMET meteorological dataset, which contains interpolated weather station data aggregated to daily summaries and downscaled to a 4 km grid.^14^ All processing and summarization of the meteorological grids takes place in the cloud using Google Earth Engine (GEE),^15^ and the user downloads daily county-level summaries. The app includes a graphical user interface that displays the raw meteorological data and allows the user to specify a date range and location to download. Code to implement this app in GEE is provided with the ArboMAP distribution, or it can be accessed directly at (https://dawneko.users.earthengine.app/view/arbomap-gridmet).

### Forecasting models

ArboMAP uses generalized additive models (GAMs) that predict whether a county will have one or more human WNV cases in a week. These are implemented as “big additive models,” computationally efficient GAMs designed to work with large datasets, using the bam() function^16^ from the R mgcv library.^17^ Predictors include mosquito infection indices and meteorological variables summarized as distributed lags, where the lagged effects are modeled as smoothed functions of the number of days before the current week. Maximum lag length is a user specified parameter and varied from 151 days in Michigan and South Dakota to 181 days in Louisiana and Oklahoma. Several options are available for model specification, including (1) different indices for summarizing the mosquito infection data, (2) different combinations of environmental variables, (3) untransformed environmental data versus environmental anomalies, (4) a single, fixed set of distributed lags versus time-varying lags that change over the course of the WNV season, and (5) different spline functions for modeling the smoothed responses. Multiple models can be combined to generate predictions based on model ensembles. Model selection is carried out using an information theoretic approach in which alternative models are compared using Akaike’s Information Criterion.^10^

To predict WNV cases during the current year, the models are first calibrated using data from previous years. Then, all available current-year environmental and mosquito data are used to generate predictions for every week of the transmission season, including backcasts for past weeks and forecasts for future weeks. Generating backcasts as well as forecasts is essential because there are delays in the diagnosis and reporting of WNV cases, and the reported numbers of cases from recent weeks are usually incomplete. Predictions are validated by calibrating the model with historical data and comparing predictions of human case occurrence to observations that were not used in the fitting process.^11^

### User interactions

ArboMAP settings are controlled by parameters, with default values provided in the R markdown script. The parameters determine how the models will be implemented, specify the time periods of historical data used for model calibration and the current-year data used for forecasts, and indicate how results will be presented. This script can be directly edited and run in RStudio, or a small R script can be run to invoke a graphical user interface (GUI) using the built-in Shiny interface. The GUI can then be used to modify the parameters and launch ArboMAP. The software automatically calibrates the models, uses them to generate forecasts, and produces formatted results in HTML or PDF format.

## Results

### Forecast outputs

**Figure 2.**
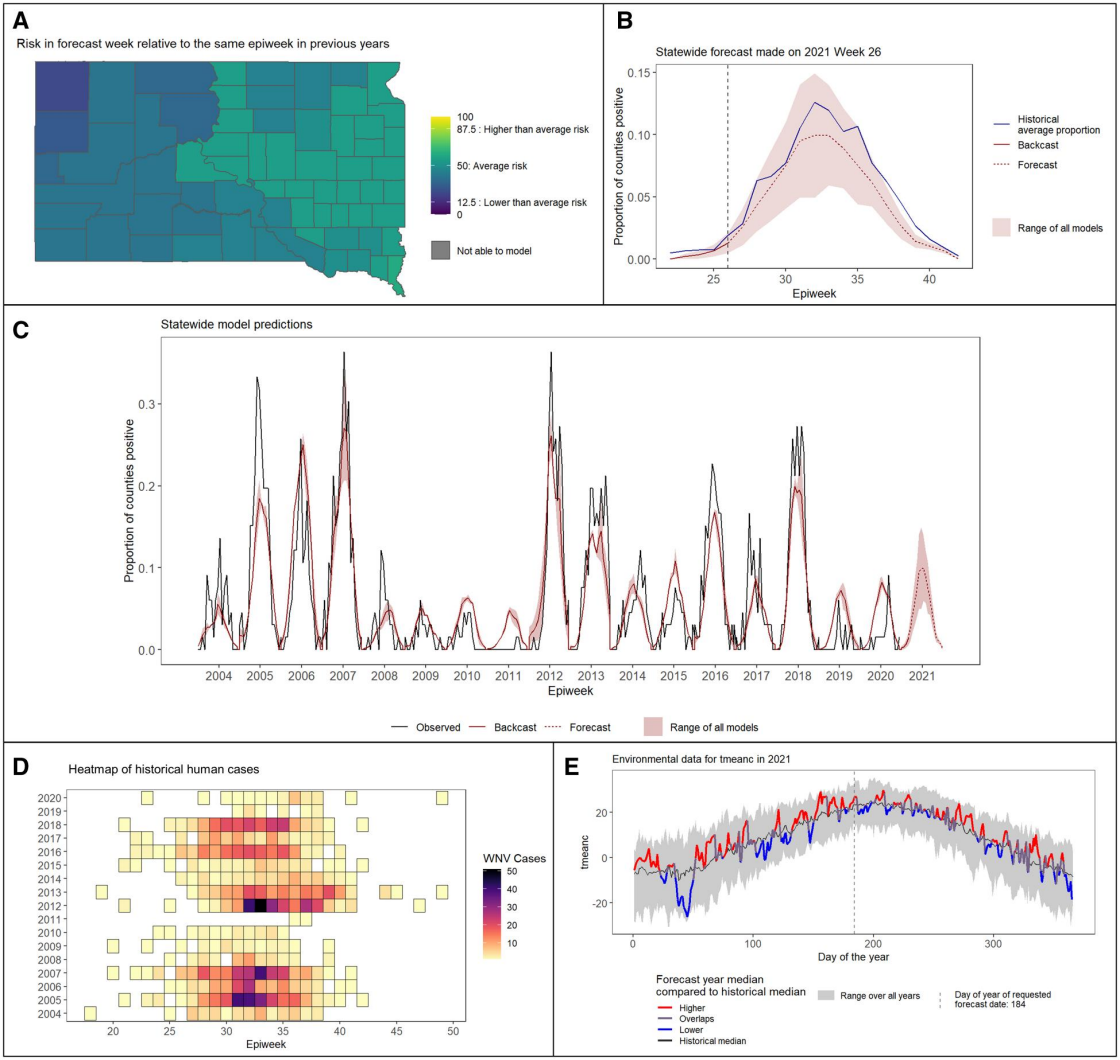
Examples of charts from an ArboMAP report for 2021 week 26 in South Dakota. (A) The relative risk of a county having at least one positive human West Nile virus. (B) The modeled epidemiological curve for the current year, including backcasts (historical predictions prior to the current week) and forecasts (future predictions after the current week). (C) Modeled epidemiological curves for all years, including fitted values in historical years (2004-2020) and forecasts for the current year (2021). (D) The weekly proportions of counties with at least one human case from historical years. (E) Daily temperatures in the current year compared to historical averages.

Model outputs are presented in a formatted report that was co-developed with partners in state health departments (Figure 2). Because of the large amount of information, it is essential to present the most important components at the beginning where they are accessible. Thus, forecast results are provided first followed by summaries of the input data and an optional appendix containing diagnostic information about the models. The forecast results section includes predictions for the current week followed by summaries of forecasts and backcasts over the entire transmission season and comparisons of the current year predictions with the historical time series. When more than one model is used, only the ensemble mean of the predictions is presented in the forecast results section for simplicity. However, details on the individual models are available in the appendix and other ensemble metrics such as the median can also be calculated. Most of the outputs are shown as maps or graphs for ease of interpretation and communication (Figure 2). Descriptive text is provided throughout to aid in interpreting the results.

### Operational use

Before the beginning of the WNV transmission season, the data on human cases, mosquito infection, and meteorological variables must be brought up to date for all previous years. These historical data are used by ArboMAP for model calibration during the upcoming year. Decisions must also be made about the types of models and the predictor variables that will be used in the WNV forecasts. Evaluations of model fit and validations of model predictions in previous years can be conducted to inform these decisions.

The ArboMAP software was designed to minimize the number of manual steps required for a weekly forecast (Figure 1). First, new mosquito data collected since the previous forecast are obtained by querying the organization’s surveillance database. (Step 1a). A mosquito data template is provided with the ArboMAP software, and a single CSV file containing all mosquito data for the current year is copied to the mosquito data folder in the ArboMAP RStudio project (Step 1b). Then, new environmental data are obtained from the ArboMAP GEE app (Step 2a). The results are downloaded as CSV files that are copied directly into the environmental data folder in the ArboMAP RStudio project and automatically ingested and combined by the software (Step 2b). The user can now start the ArboMAP application and modify default parameters using the GUI (Step 3a). In most cases, the same set of parameters are used to generate forecasts throughout an entire season and the only required change is the date of the current forecast week. At this point, the run is initiated, and the modeling and report generation (Step3b) are automated.

An earlier version of ArboMAP was first implemented by the South Dakota Department of Health in 2016, and the tool has been used there since. Following several years of collaboration with the developers, the Louisiana Department of Health began using ArboMAP independently in 2022. Southern Nazarene University collaborated with the Oklahoma City County Health Department to generate forecasts beginning in 2022. The Michigan Department of Health and Human Services began generating forecasts with ArboMAP in 2023. In South Dakota and Michigan, ArboMAP forecasts have been incorporated into online WNV dashboards and communicated with stakeholders via statewide email listservs. Because reported human cases are often delayed by weeks or months and observed mosquito abundance is a poor indicator of transmission risk, predictions from ArboMAP have been useful for highlighting WNV risk and targeting mosquito control and disease prevention activities prior to the seasonal peak in transmission.^11^

## Discussion

There is considerable interest in developing and testing new approaches for modeling and forecasting outbreaks of WNV and other infectious diseases.^18–21^ If these techniques are combined with improved systems for timely and accurate collection of relevant data, they have the potential to improve public health responses to outbreaks.^22^ The importance of having robust software to operationalize disease early warning systems has been recognized,^23^ but this topic has not been widely addressed in the scientific literature.^24^ The ArboMAP software system has been successfully implemented for routine forecasting of WNV. It can be used to forecast WNV in other locations where sufficient data are available and could also be adapted to work with other climate-sensitive vector-borne diseases.

The design of ArboMAP represents a compromise in which most of the time-consuming steps required for data processing and harmonization, model fitting and prediction, and presentation of the forecast results have been automated. Other aspects of the software, such as the connections to external databases, have been implemented as loose couplings and require additional manual steps for data acquisition. ArboMAP was developed as a client-side application that is installed on a laptop or desktop workstation rather than a cloud-based application that can be remotely accessed. These decisions make it practical for multiple public health institutions to independently use ArboMAP. Because of the security and privacy issues associated with health surveillance data, it was not feasible for us to develop a tight coupling solution for connecting ArboMAP with these systems. These issues also limited our options for accessing surveillance data through the cloud. Although the design of ArboMAP has facilitated its use in multiple states, opportunities remain to further automate the process of data acquisition and decrease the user effort required to generate forecasts.

Co-development with public health partners has been essential in designing the ArboMAP system. The current forecasting reports were informed by design and evaluation workshops held in 2021 and 2022. Key design principles included emphasizing visualizations over written text, creating stand-alone figures that can be copied to other reports or websites to communicate the forecasts, using consistent color schemes and formatting throughout the report, and adjusting the order so that the most important results can be found on the first few pages of the report. User feedback has also informed the development of the user-specified parameters and the design of the GUI. All ArboMAP code along with comprehensive documentation and artificial datasets for demonstration and testing are available on GitHub (https://github.com/EcoGRAPH/ArboMAP). Users can run the software with their own datasets or customize the code to meet specific needs. Potential changes could include integrating novel streams of data, incorporating different predictive modeling techniques, or modifying the information provided in the forecasting reports.

## Supplementary Material

ooad110_Supplementary_Data

## Data Availability

Public health surveillance data on human West Nile virus cases and mosquito infection are collected and maintained by state departments of health and are not publicly distributable. Meteorological data from GridMET are publicly available at https://www.climatologylab.org/gridmet.html. A Google Earth Engine app for accessing and downloading the GridMET data summarized by county can be accessed at https://dawneko.users.earthengine.app/view/arbomap-gridmet.
