# Supplementary material for: The Arbovirus Mapping and Prediction (ArboMAP) system for West Nile virus forecasting
Source: JAMIA Open. 2023 Dec 21;7(1):ooad110. doi: 10.1093/jamiaopen/ooad110 (PMC10766066; doi:10.1093/jamiaopen/ooad110)
Supplement: ooad110_Supplementary_Data [file ooad110_supplementary_data.docx]

# Forecasting Models

ArboMAP uses an ensemble of mathematical models that each predict if a county will report at least one human West Nile virus (WNV) case in a given week (a ‘positive county-week’). These models are fitted to the data as binomial big additive models run with the bam() function from the mgcv library in R^1^. Predictor variables include weather variables with effects modeled as distributed lags, and the mosquito infection growth rate (MIGR). The distributed lag approach models the effect of the independent variable as a smoothed function of lag date using a thin-plate spline. Technical details of the distributed lag approach are provide in Gasparrini et al.^2^.The MIGR estimates the rate of rate of amplification in mosquitoes and birds during the early part of the transmission season. Detailed explanation of the formulation of the MIGR index is provided by Davis et al.^3^. The basic model form is:

$$\mathrm{logit}\left( p_{i,t} \right)=c_{0i}+c_{1}m_{i,t}+cyc\left( w \right)+\sum_{k=1}^{2} \sum_{l=0}^{L} s_{k}\left( l \right)\mu_{i,t-l}$$

where *p_i,t_* is the probability of one or more WNV cases in county *i* at time *t*, *c_0i_* is a county-level fixed effect, *m_i,t_* is the MIGR in county *i* at time *t*, *c_1_* is the parameter for MIGR, *cyc(w)* is a cyclical seasonal function, *k* indexes two meteorological variables, *l* indexes daily lags up to *L*=120 days in the past, *s_k_(l)* is a thin-plate spline function of lag days, *u_i, t-l_* is a meteorological variable for county *i* at time *t-l*. The cyclical seasonal term is optional. If it is included, then the *u_i, t-l_* are anomalized meteorological variables calculated by subtracting long-term daily mean from each daily observation. If it is not included, then the *u_i, t-l_* are untransformed meteorological variables.

A second variant of the model allows the distributed lag effects to vary throughout the transmission season. This implementation allows the strength and lag period of environmental predictors to be different in the spring when amplification occurs, the summer when human transmission peaks, and in the fall when human transmission declines. This seasonally varying model form is:

$$\mathrm{logit}\left( p_{i,t} \right)=c_{0i}+c_{1}m_{i,t}+cyc\left( w \right)+\sum_{k=1}^{2} \sum_{l=0}^{L} s_{k}\left( l,w \right)\mu_{i,t-l}$$

where *s_k_(l, w)* is a thin-plate spline function of lag days and week of the year and all other variables are as described previously. Additional information about the underlying forecasting models is provided by Davis et al.^3^ and Wimberly et al.^4^

# Model Fitting and Validation

Forecasting models can have different model forms, including fixed versus seasonally varying distributed lags and untransformed versus anomalized environmental variables. Models can also include different combination of environmental predictors such as mean, minimum, or maximum temperature, precipitation, relative humidity and vapor pressure deficit. In addition, there are a variety of other options that can be specified such as the number and locations of strata used in the MIGR algorithm, the maximum distributed lag length, and the beginning and end dates that can be modified by the user. Understanding model sensitivity to these choices requires quantitative assessment of their impacts on model fit. Model selection for ArboMAP is carried out by comparing alternative model parameterizations using an information theoretic approach based on the Akaike’s Information Criterion (AIC) statistic. Selection based on AIC helps to avoid overparamerization because the statistic is penalized for the number of model parameters. A case study of model selection for South Dakota is included in Davis et al.^3^

The ArboMAP forecasting report includes provides an AIC statistic for each model that is based on calibration to the historical data. It also includes time series graphs of the fitted values and the historical observations that allow users to visualize the degree of model fit. ArboMAP can be configured to fit multiple model forms in a single run, and different Individual ArboMAP runs can be configured to use different combinations of environmental variables. Thus, end users have the capability to carry out model selection and fit assessment.

Model validation can be conducted retrospectively or prospectively. Retrospective validation involves running the model for prior years using only the data that would have been available at a particular forecast week. The predictions are then compared to observed values that are outside the scope of the model calibration. Such an analysis has been carried out for South Dakota to assess model accuracy from 2016-2019^4^. Prediction accuracy was compared for several different models, including 1) a baseline models that only included seasonal trends and county-level means, 2) a model based only on the MIGR, 3) models based only on meteorological predictors, and 4) combined models based on MIGR and mosquito predictors. A key finding was that the combined models performed the best, with higher accuracy than the baseline model and models based only on MIGR or environmental data. Model predictions distinguished between years with high and low WNV case counts, and that peak accuracy was achieved in late June, providing sufficient time for public health response prior to the peak of human cases in early August.

Prospective model validation is carried out by comparing operational predictions of weekly and annual case counts with the human WNV surveillance data when it is finalized after the end of the transmission season. For example, a prospective validation of WNV forecasting in South Dakota in 2016 found that forecasts made in that year accurately predicted an upsurge in cases compared to the previous years and also captured an earlier-than-usual increase in human cases^5^.

# Accessing and Running ArboMAP

The ArboMAP software is available on a GitHub at <https://github.com/EcoGRAPH/ArboMAP>. Detailed documentation are provided, including a general user guide^6^ as well as “quick-start” guides for annual updates and weekly forecasts. The archive also includes synthetic example data that can be used to run a model demonstration. To run the demonstration, the user must have the latest versions or R and RStudio installed on their computer. The entire archive can be downloaded and copied onto the user’s file system. The user can double-click on the ArboMAP.Rproj file to open the project in RStudio and run ArboMAP_run_forecast_html.R. Running this file will open a brower window through which the user can select various options and click Save to start the run. Once the run has been started, the menu will be grayed out and the user should switch back to the RStudio console to monitor the progress of the run. Depending on the available computer resources, the size of the dataset, and the numbers of types of models selected, the run may take between 5-10 minutes. When the run is completed, the HTML report will be generated in a browser page. The report can also be generated as PDF if appropriate software (MiKTeX or TinyTeX) is installed.

# References

1. *mgcv*. 2015. h
<ttp://CRAN.R-project.org/package=mgcv>

2. Gasparrini A, Armstrong B, Kenward MG. Distributed lag non‐linear models. *Stat Med*. 2010;29(21):2224-2234.

3. Davis JK, Vincent GP, Hildreth MB, Kightlinger L, Carlson C, Wimberly MC. Improving the prediction of arbovirus outbreaks: A comparison of climate-driven models for West Nile virus in an endemic region of the United States. *Acta Trop*. 2018;185:242-250.

4. Wimberly MC, Davis JK, Hildreth MB, Clayton JL. Integrated Forecasts Based on Public Health Surveillance and Meteorological Data Predict West Nile Virus in a High-Risk Region of North America. *Environ Health Persp*. 2022;130(8):087006.

5. Davis JK, Vincent G, Hildreth MB, Kightlinger L, Carlson C, Wimberly MC. Integrating environmental monitoring and mosquito surveillance to predict vector-borne disease: prospective forecasts of a West Nile virus outbreak. *PLoS Curr*. 2017;May 23, 9(May 23)

6. Nekorchuk DM, Davis JK, Wimberly MC. *ArboMAP User Guide*. 2023. <https://github.com/EcoGRAPH/ArboMAP>
